# Supplementary material for: A Likelihood Approach for Real-Time Calibration of Stochastic Compartmental Epidemic Models
Source: PLoS Comput Biol. 2017 Jan 17;13(1):e1005257. doi: 10.1371/journal.pcbi.1005257 (PMC5240920; doi:10.1371/journal.pcbi.1005257)
Supplement: S1 File — (TAR.GZ) [file pcbi.1005257.s014.tar.gz › HSPH_Online-SI-Revision/PF08/n100-severe/n100-severe_PF2_summary2.pdf]

peak -8	3w spec	3w cum	5w spec	5w cum	end	3w spec LOG	3w cum LOG	5w spec LOG	5w cum LOG	end LOG
mean	75.3	69.9	74.6	70.7	25.2	0.4	0.3	0.7	0.5	0.1
median	61.3	52.6	73.4	66.7	23.4	0.4	0.3	0.7	0.5	0.1
quantile .1	39.4	32.	45.5	39.7	12.	0.2	0.2	0.3	0.2	0.
quantile .9	154.4	149.3	103.7	113.6	39.7	0.6	0.5	1.	0.2	0.2
19	26	14	16	31						

peak -4	3w spec	3w cum	5w spec	5w cum	end	3w spec LOG	3w cum LOG	5w spec LOG	5w cum LOG	end LOG
mean	30.	28.5	22.4	23.7	47.1	0.2	0.2	0.1	0.1	0.2
median	23.6	21.	18.1	18.4	45.5	0.1	0.1	0.1	0.1	0.2
quantile .1	5.	4.8	5.2	5.6	27.1	0.	0.	0.	0.	0.1
quantile .9	60.3	59.4	43.5	55.2	67.	0.4	0.4	0.2	0.	0.2
10	9	12	11	0						

peak	3w spec	3w cum	5w spec	5w cum	end	3w spec LOG	3w cum LOG	5w spec LOG	5w cum LOG	end LOG
mean	104.1	37.7	386.	64.6	133.6	0.3	0.1	0.6	0.2	0.4
median	79.9	29.8	222.1	55.2	121.1	0.3	0.1	0.5	0.2	0.3
quantile .1	20.8	10.5	59.4	19.3	67.3	0.1	0.	0.2	0.1	0.2
quantile .9	204.1	75.1	961.3	111.8	203.6	0.5	0.2	1.	0.1	0.5
0	1	0	0	0						

peak +4	3w spec	3w cum	5w spec	5w cum	end	3w spec LOG	3w cum LOG	5w spec LOG	5w cum LOG	end LOG
mean	1289.4	565.9	5029.4	710.3	966.1	0.9	0.7	1.1	0.7	0.9
median	539.2	325.3	1021.6	407.4	585.	0.8	0.6	1.	0.7	0.8
quantile .1	112.4	81.4	173.3	97.4	142.3	0.3	0.3	0.4	0.3	0.4
quantile .9	2392.4	1432.2	11 997.2	1823.	2476.6	1.4	1.2	2.1	0.3	1.4
0	0	0	0	0						

peak +8	3w spec	3w cum	5w spec	5w cum	end	3w spec LOG	3w cum LOG	5w spec LOG	5w cum LOG	end LOG
mean	136 812.	8452.6	146 619.	11 001.2	14 456.2	1.5	1.2	1.8	1.3	1.4
median	1876.4	1290.4	3385.1	1553.2	2057.3	1.3	1.1	1.5	1.2	1.3
quantile .1	227.	183.6	330.9	203.6	274.2	0.5	0.5	0.6	0.5	0.6
quantile .9	55 883.7	19 084.7	856 470.	25 319.2	33 368.9	2.7	2.3	3.9	0.5	2.5
0	0	0	0	0	{}	{}	{}	{}	{}	{}
